# The Role of Obstructive Sleep Apnea in Pulmonary Hypertension Associated with Lung Diseases (Group 3 Pulmonary Hypertension): A Narrative Review

**DOI:** 10.3390/jcm14155442

**Published:** 2025-08-01

**Authors:** Athiwat Tripipitsiriwat, Atul Malhotra, Hannah Robertson, Nick H. Kim, Jenny Z. Yang, Janna Raphelson

**Affiliations:** 1 Division of Pulmonary, Critical Care, Sleep Medicine and Physiology, Department of Medicine, University of California San Diego, San Diego, CA 92037, USA; amalhotra@health.ucsd.edu (A.M.);; 2 Division of Respiratory Disease and Tuberculosis, Department of Medicine, Faculty of Medicine Siriraj Hospital, Mahidol University, Bangkok 10700, Thailand

**Keywords:** sleep-disordered breathing, hypoxemia, overlap syndrome, volume overload, cor pulmonale, GLP-1, cardiovascular disease

## Abstract

Obstructive sleep apnea (OSA) could increase pulmonary artery pressure. However, the clinical consequences vary, mainly depending on comorbidities. Patients with pulmonary hypertension associated with lung diseases (World Health Organization (WHO) Group 3 pulmonary hypertension) are particularly vulnerable increases in pulmonary artery pressure. Managing pulmonary hypertension in this specific patient population presents a considerable challenge. While positive airway pressure therapy for OSA has shown promise in improving pulmonary hemodynamics in patients with obesity hypoventilation syndrome and chronic obstructive pulmonary disease, evidence is lacking for similar improvements in those with other pulmonary diseases and hypoventilation disorders. Furthermore, pulmonary-artery-specific therapies may carry a risk of clinical worsening in this group. Weight management and new pharmacotherapy have together emerged as a crucial intervention, demonstrating benefits for both OSA and pulmonary hemodynamics. We reviewed key studies that provide insights into the influence of OSA on WHO Group 3 pulmonary hypertension and the clinical management of both conditions.

## 1. Introduction

Obstructive sleep apnea (OSA) is a widely recognized condition that exacerbates cardiovascular disease, including pulmonary hypertension (PH). The number of affected individuals is growing, particularly due to the obesity pandemic [1]. However, the clinical impact of OSA is remarkably heterogeneous, with multiple OSA endotypes having been described [2]. This variability might explain the difficulty in demonstrating the effectiveness of various treatment strategies in prospective clinical studies despite compelling anecdotal clinical successes [3]. A particular population of interest is individuals with chronic lung diseases who are at risk of profound ventilatory derangements during sleep and already vulnerable to the development of pulmonary hypertension and right heart failure (World Symposium on Pulmonary Hypertension (WSPH) or World Health Organization (WHO) Group 3 pulmonary hypertension). OSA has long been thought to worsen cardiovascular prognosis in this group and may be an important contributor to PH progression and mortality [4,5,6,7].

Decades of research have importantly advanced our understanding of the complex interplay between OSA and PH. This review aims to highlight key studies that have clarified the link between OSA and WHO Group 3 pulmonary hypertension. Furthermore, we will critically evaluate the effects of treatment in this high-risk patient group.

## 2. Updates in WHO Group 3 Pulmonary Hypertension

Since the 2nd WSPH in 1998, Group 3 pulmonary hypertension has been designated as pulmonary hypertension associated with lung diseases and/or hypoxia, including sleep-disordered breathing and hypoventilation disorders [8,9]. Over time, the clinical classification of PH has been revised and updated in response to evolving knowledge regarding its pathophysiology, clinical presentation, and therapeutic considerations within each group. Three notable recent changes might affect ongoing research and clinical practice.

First, as of the 6th WSPH in 2018, and later adopted by the European Cardiology Society and European Respiratory Society (ECS/ERS) 2022 guidelines for the diagnosis and treatment of pulmonary hypertension, sleep-related breathing disorders have been removed from the subclassification of Group 3 pulmonary hypertension [10]. (We discussing this matter further in a section in this article “Obstructive Sleep Apnea as a Primary Etiology of Pulmonary Hypertension”).

Next, at this same symposium, the hemodynamic definition of pulmonary hypertension was adjusted—newly defined as a mean pulmonary arterial pressure (mPAP) of 20 mmHg, compared to the previously accepted threshold of 25 mmHg, and a pulmonary vascular resistance (PVR) of 2 Wood units (WU), compared to the prior 3 WU [10,11]. This change increased the number of PH diagnoses in patients with respiratory disease. For example, the prevalence of PH associated with chronic obstructive pulmonary disease (COPD) increased from 52.4% to 82.4% with the 6th WSPH definition [12]. This change might affect the magnitude of the association between sleep-related breathing disorders and pulmonary vascular disease. Investigations using new hemodynamic definitions and updated imaging techniques, such as cardiac MRI, may also provide new insights into the interaction of sleep-related breathing disorders and pulmonary hemodynamics.

Third, the definition of Group 3 PH is currently diagnosis-oriented rather than treatment-oriented. Previous versions of the consensus mainly relied on physiological abnormalities by spirometry [13]. In contrast, the latest 7th WSPH has suggested using the clinical diagnosis (Table 1). This change underscores the significance of various parenchymal abnormalities and their pathophysiological contributions to the degree of PH in this patient group. This heterogeneity may explain the variable results in studies depending on each primary lung disease diagnosis. Thus, extrapolating the effectiveness of OSA treatment requires judicious consideration, especially between entities with and without associated lung parenchymal abnormalities.

## 3. Obstructive Sleep Apnea and Patients with Pulmonary Hypertension Associated with Pulmonary Diseases (WHO gr. 3 PH)

One early possible description of the association of obstructive sleep apnea (OSA) with pulmonary hypertension (PH) was in The Pickwick Papers by Charles Dickens in which a character called Fat Boy Joe was described. Dickens portrayed an obese, excessively sleepy errand boy who snored in his sleep. Of note, Fat Boy Joe had “dropsy”, which was a description of peripheral edema at that time and a plethoric face, which were suggestive of cor pulmonale and right-sided heart failure [15]. While the literary description may not be sufficiently detailed to diagnose accurately whether Fat Boy Joe had obesity hypoventilation syndrome (OHS) rather than OSA, it has inspired researchers to investigate the role of sleep apnea in contributing to pulmonary hypertension and right heart failure.

The association between sleep apnea and pulmonary hypertension was previously anecdotal until the introduction of methods to assess pulmonary vascular hemodynamics including right heart catheterization (RHC). Various studies have shown that obstructive apnea could cause an acute increase in pulmonary artery pressure (PAP) [16]. However, the magnitude of the increase was modified by various factors, particularly the degree of baseline hypoxemia and cardiac function, cardiopulmonary interactions induced by intrathoracic pressure swings, and sleep-stage-dependent changes [17,18]. The elevation of pulmonary artery pressure in OSA is thought to be at least partially attributable to hypoxic pulmonary vasoconstriction [18,19].

Furthermore, among patients with chronic lung disease, the increased propensity to develop hypercapnia might be another factor that enhances the effect of hypoxic vasoconstriction. Acute hypercapnia can contribute to pulmonary vasoconstriction, which is likely to occur with repetitive apnea in OSA [20]. Chronic hypercapnia also significantly affects pulmonary vasculature responses in the presence of hypoxemia [21]. Clinical studies investigating patients with chronic lung disease who experience diurnal elevation in PAP have suggested that OSA may contribute to the severity of PH, particularly in the context of daytime abnormalities in blood gases [22,23]. Similarly, clinical data have shown that patients with obesity and daytime hypercapnia (obesity hypoventilation syndrome) have a higher prevalence of pulmonary hypertension compared to those without hypoventilation [24]. Control of OSA in patients with OHS results in a reduction in systolic PAP and mortality in these patients comparable to non-invasive ventilation (NIV) use [25,26].

Separate from OSA, patients may also have elevated PAP due to lung parenchymal pathology. The presence of OSA might exacerbate the elevation of PAP in this group of patients. One condition that highlights this finding is chronic obstructive pulmonary disease (COPD). Patients with overlap syndrome (concurrent OSA and COPD) have marked thickening of the right ventricle and have worse survival [4].

There is a paucity of data on idiopathic pulmonary fibrosis (IPF) and OSA, particularly the effect on pulmonary hemodynamics [7]. A small study showed that the degree of sleep desaturation in IPF correlated with PAP, the apnea–hypopnea index (AHI), and survival [27]. Another study in a more general patient population with fibrotic interstitial lung disease (IPF and non-IPF fibrotic ILD) also found an association between nocturnal desaturation and various markers of pulmonary hypertension, such as tricuspid regurgitation velocity, pulmonary artery diameter, and the presence of PH identified on echocardiogram [28]. Nevertheless, whether the degree of nocturnal hypoxemia was primarily from the severity of OSA or ILD remains inconclusive. A recent prospective study in fibrotic ILD patients using WatchPAT200^®^ (Itamar Medical, Caesarea, Israel) found that 19 out of 32 (59.3%) subjects with OSA did not have significant nocturnal hypoxemia (defined by having oxygen saturation less than 90% for more than 10% of the total sleep time) and 7 out of 20 (35%) subjects with nocturnal hypoxemia did not have OSA [29]. Interestingly, the abnormalities in pulmonary function tests (PFTs), disease severity score, and baseline echocardiogram PH probability were not different across the group of subjects with and without nocturnal hypoxemia or OSA. This finding suggests that OSA might not be the primary cause of nocturnal hypoxemia in this population. However, the treatment of OSA may still be emphasized as a reversible cause of nocturnal hypoxemia. Of note, the trajectory of pulmonary artery hemodynamics was unknown, as the data were not reported in this study.

Finally, in some cases, impaired pulmonary hemodynamics can also be attributed to OSA exacerbating comorbid cardiovascular disease (CVD). OHS, COPD, and IPF patients have a high prevalence of CVD, including metabolic syndrome and aging-related disease, which are associated with left-sided heart dysfunction [13]. Various neuromuscular disorders causing hypoventilation (e.g., Duchenne/Becker muscular dystrophy, limb-girdle muscular dystrophies, myotonic dystrophies) also have comorbid cardiomyopathies [30].

**Figure 1:**
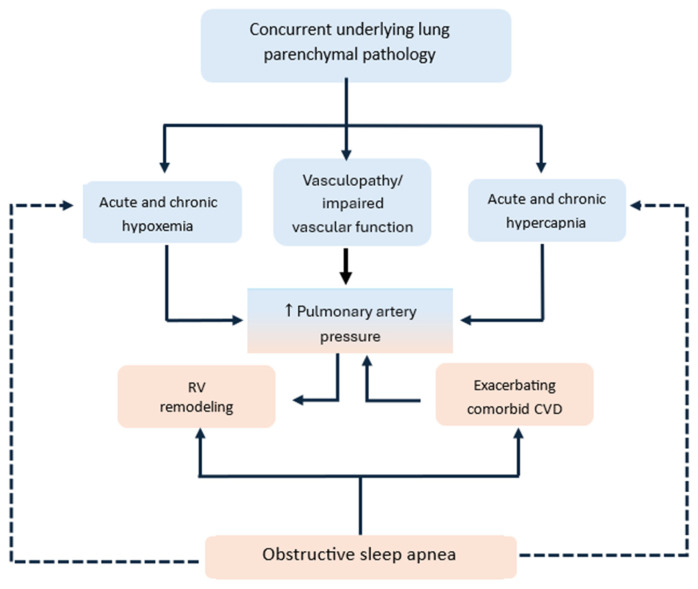
Influence of obstructive sleep apnea on pulmonary artery pressure and cardiopulmonary disease. Dotted lines represent indirect relationships. Abbreviations: RV: right ventricle, CVD: cardiovascular disease.

Overall, patients with WHO Group 3 pulmonary hypertension are susceptible to further elevations of PAP induced by OSA (Figure 1). These patients suffer more severe degrees of nighttime hypoxemia and/or hypercapnia due to poorer gas exchange capacity at baseline as well as higher baseline PAP from lung pathology and associated cardiac comorbidities. The guidelines strongly recommend optimizing the treatment of sleep-disordered breathing in this patient group [11].

## 4. Obstructive Sleep Apnea as a Primary Etiology of Pulmonary Hypertension

Whether obstructive sleep apnea solely results in pulmonary vascular disease remains a debatable concept. The evidence of pulmonary vascular remodeling due to hypoxia has been seen in various preclinical models but not verified in human studies [31]. In human clinical studies, echocardiography is frequently used as the primary tool to identify pulmonary hypertension (PH). However, echocardiographic data alone presents challenges in distinguishing the etiology of elevated pulmonary artery pressure, particularly in differentiating between left-sided heart dysfunction and intrinsic changes within the pulmonary arteries. Despite these challenges, several studies have attempted to characterize the alterations in pulmonary pressure associated with OSA.

Notably, Sajkov and McEvoy have published a series of papers investigating the presence of daytime pulmonary hypertension (mPAP > 20 mmHg) in patients with OSA [31,32,33,34]. The authors prospectively included OSA patients without prior cardiopulmonary disease and evaluated them using Doppler echocardiography to estimate the mPAP with an investigator-developed algorithm. In this context, they observed a PH prevalence of 34% and three other important observations [33]. First, the PH was generally mild to moderate (mPAP 20–31 mmHg). Second, the authors observed various physiological features suggesting pulmonary vascular remodeling: marked hypoxic vasoreactivity, exaggerated elevation in PAP with dobutamine infusion, and a higher degree of small airway closure demonstrated in lung function testing (increased closing volume). These findings suggest that some patients with OSA and elevated PAP might be vulnerable to PAP elevation in particular situations, including acute pneumonia, high altitude, etc. Third, the authors observed an improvement in pulmonary hemodynamics within 4 months of CPAP therapy, suggesting that OSA may be one of the few reversible causes of pulmonary hypertension [34].

Subsequent similar studies conducted in patients with OSA without major pulmonary disease have indicated a prevalence of PH ranging between 12% and 60% (mPAP cut-off was 20 or 25 mmHg depending on the study) [35]. Although these studies might overestimate the prevalence of PH in OSA due to selection bias and the inability to rule out subclinical left-sided heart failure due to the lack of right heart catheterization data, they highlighted the high prevalence of PH in OSA.

Interestingly, there have been more studies conducted to investigate RV function in OSA; these studies have shown evidence of RV remodeling in this population. A post hoc analysis of the Framingham Heart Study found increased RV thickness on echocardiogram in patients with an increased respiratory disturbance index without differences in comorbidities (e.g., hypertension and obesity) and left heart function [36]. Another recent cross-sectional study found that OSA was associated with RV dilation and hypertrophy, even after adjusting for body mass index (BMI), left heart dimension, and the presence of PH (systolic PAP ≥ 40 mmHg) [37]. More advanced echocardiographic or MRI parameters of right heart function showed signals toward increased abnormalities in patients with OSA; however, the findings are quite variable. A comprehensive review of OSA and right ventricular remodeling has been published [38].

Another important aspect is the impact of PH in OSA patients. A study conducted by Minai et al. is the only study to our knowledge that explicitly provided both right heart catheterization and survival data in an OSA population: the survival rates at 4 and 8 years were 90% and 76% in patients without PH versus 75% and 43% in patients with PH [39]. The study revealed that OSA patients with PH (mPAP ≥ 25 mmHg) had a worse prognosis regardless of the presence of left-sided heart disease. Furthermore, it is now widely acknowledged that even mild elevations in mPAP (>20 mmHg) have been associated with increased mortality [40].

However, due to the paucity of the evidence supporting isolated OSA causing significant PH, the latest clinical practice guideline endorsed by ESC/ERS and WSPH removed sleep-disordered breathing as a cause of Group 3 pulmonary hypertension [11]. The impact of this change remains unclear. Nevertheless, the aforementioned studies do support that some patients with OSA could have elevated PAP. While the increase in PAP may not be clinically significant at rest, it may impact the individual’s capacity to tolerate exercise and conditions leading to acute hypoxemia (e.g., pneumonia) [41]. Further studies are needed to determine reasonable screening methods and to evaluate the impact of elevated PAP in OSA patients.

## 5. Effects of Therapeutic Strategies on Pulmonary Artery Pressure and Obstructive Sleep Apnea in WHO Group 3 PH

Patients with WHO Group 3 PH need holistic treatment that includes specific management for each underlying primary lung disease [13]. This treatment approach also encompasses a wide array of general measures for managing associated comorbidities, nutrition, infection prevention and rehabilitation [42]. We will discuss treatment strategies for OSA and other sleep-disordered breathing conditions, as well as their effects on the trajectory of pulmonary hemodynamics (Table 2).

### 5.1. Positive Airway Pressure Therapy

Continuous positive airway pressure (CPAP) can improve pulmonary hemodynamics during follow-up of patients with isolated (eucapnic) OSA [34], and a meta-analysis confirmed that CPAP in this group was associated with a significant reduction in PAP of 13.3 mmHg [43]. In patients with hypoventilation, the efficacy of CPAP has been primarily investigated in OHS and is associated with increased survival [44]. The largest trial, conducted by the Pickwick study group, showed that CPAP use could significantly decrease systolic pulmonary artery pressure (SPAP) (average SPAP decreased from 40.5 mmHg to 35.3 mmHg), comparable to non-invasive ventilation (NIV) in OHS patients with severe OSA (average SPAP decreased from 41.5 mmHg to 35.5 mmHg) [25]. This effect was observed in both short-term and long-term use of CPAP, extending up to three years [25]. CPAP has not been systemically investigated in other causes of hypoventilation to our knowledge.

Another group of patients with lung disease for whom CPAP showed survival benefits is patients with COPD and OSA (overlap syndrome) [4,45]; however, the benefit of CPAP in patients with overlap syndrome who also have diurnal hypercapnia is uncertain [46]. Moreover, the data regarding pulmonary hemodynamic improvement with CPAP in patients with overlap syndrome have not been reported.

Bilevel-positive airway pressure (BIPAP) is a cornerstone of neuromuscular and thoracic restrictive disease management. Both invasive and non-invasive ventilation are associated with reduced mortality, improved quality of life, and enhanced sleep quality [47]. However, the effect on pulmonary artery pressure is typically not evaluated in this patient group. As mentioned above, BIPAP has been shown to improve pulmonary artery pressure in OHS. The clinical benefits of BIPAP for each disorder are beyond the scope of this review but are available in the cited literature.

To our knowledge, no studies have systematically studied the effects of non-positive airway pressure therapy (e.g., mandibular advancement device, upper airway surgery) for OSA to pulmonary hemodynamics.

### 5.2. Oxygen Therapy

Patients suffering from chronic pulmonary diseases accompanied by severe daytime hypoxemia (PaO_2_ < 55 mmHg) or moderate hypoxemia (PaO_2_ 55–59 mmHg) with symptoms of right heart failure are typically prescribed long-term oxygen therapy (>15 h/day), which has been shown to improve survival rates and pulmonary hemodynamics [48]. However, long-term oxygen therapy or nocturnal-only oxygen therapy has not demonstrated additional benefits in COPD patients with a milder degree of hypoxemia. Chaouat et al. studied the effects of nocturnal oxygen therapy in patients with COPD with mild-to-moderate hypoxemia (PaO_2_ 56–69 mmHg) and marked nocturnal desaturation [49]. This study showed nocturnal oxygen therapy did not improve pulmonary hemodynamics nor clinical progression. Subsequent randomized control trials also failed to demonstrate additional benefits of nocturnal oxygen therapy in COPD with milder hypoxemia [50,51]. Notably, clinical trials typically excluded patients who demonstrated cyclical desaturation or known OSA—a possible subgroup of patients yet to be defined who may benefit from nocturnal oxygen therapy. A randomized, sham-controlled, cross-over trial, investigating nocturnal oxygen supplementation of 2 L per minute, showed an improvement of apnea severity and nocturnal oxygenation in patients with OSA and ILD, with 71% of the patients having an AHI reduction of more than 50% (oxygen responders) [52].

An official guideline from the American Thoracic Society on home oxygen therapy recommends two strategies: long-term oxygen therapy (LTOT) for patients with COPD or ILD who have severe hypoxemia and ambulatory oxygen therapy for those with severe exertional hypoxemia (SpO_2_ ≤ 88% on exertion) [53]. However, this guideline does not make recommendations regarding LTOT for patients with features of cor pulmonale who do not have severe daytime hypoxemia. This area requires further investigation.

The efficacy of nocturnal oxygen therapy in patients with a discrepancy between the degree of daytime hypoxemia and pulmonary hemodynamics might also be worth reviewing. Sleep-related hypoxemia has recently been recognized as a risk factor for death in pulmonary arterial hypertension (PAH). A mean nocturnal oxygen saturation of less than 90%, or more than 37% of time spent with oxygen saturation below 90% (T90), was associated with increased mortality in this group [54]. A study by Ulrich et al. showed improvement in six-minute walk distance and right ventricular fractional area change with nocturnal oxygen therapy in patients with PAH or chronic thromboembolic pulmonary hypertension (CTEPH) and nocturnal hypoxemia (mean SpO_2_ < 90% or oxygen desaturation >3% index more than 10/h) [55]. It remains uncertain whether the same benefit would be observed in the subgroup of WHO Group 3 PH patients who have severe PH but do not experience severe daytime hypoxemia.

### 5.3. PAH-Targeted Drugs

PAH-targeted therapies have been studied in WHO Group 3 PH, but results have been mixed, with some even showing harm. RISE-IIP (riogiciguat in ILD) [56], ARTEMIS-IPF (ambrisentan in IPF) [57], and PERFECT (inhaled Treprostinil in COPD) [58] were terminated prematurely due to an increase in serious adverse events (e.g., disease progression, exacerbation of COPD) in the treatment group and signals toward increased mortality.

The only approved PAH-targeted therapy in Group 3 PH is inhaled treprostinil, specifically for patients with concurrent interstitial lung disease and PH (PH-ILD), as evaluated in the INCREASE study [59]. In this randomized control trial, the treatment group showed improvement in six-minute walk distance and NT-proBNP level and had fewer clinical worsening events. Although there was no analysis of the presence of sleep-disordered breathing and other sleep parameters in INCREASE, the trial did not exclude patients with sleep-disordered breathing. However, due to the potential risk of harm, PAH-targeted drugs are generally not considered in WHO Group 3 PH unless the PH is severe. Guidelines recommend that patients being considered for PH-specific drug therapy should be evaluated in an experienced center [11].

### 5.4. Lung Transplant

Generally, the presence of sleep-disordered breathing before transplant does not predict adverse effects on survival [60]. However, the prevalence and effect of sleep-disordered breathing after transplant remains a matter of ongoing study. A retrospective review found a high prevalence of sleep-disordered breathing after lung transplant, especially in COPD (71.1%) and pulmonary fibrosis (65.1%) [61]. The majority of sleep-disordered breathing was OSA (73.8%) [61]. The effects of corticosteroids on the upper airway and accumulation of fat may contribute to the incidence of sleep apnea in this population [62,63]. The authors also found that patients who use CPAP had better survival [61], although the mechanism is unclear and may be related to the healthy user effect. Another study also found a high prevalence of sleep-disordered breathing (63%) post-transplant, and its presence in this population was associated with higher blood pressure [64]. Both studies found that the Epworth sleepiness scale performed poorly in discriminating between those with or without sleep-disordered breathing [61,64]. The role of screening for OSA after lung transplantation should be investigated prospectively.

### 5.5. Pulmonary and Cardiac Rehabilitation

The effectiveness of pulmonary rehabilitation has not been directly evaluated in WHO Group 3 PH populations to our knowledge. Nevertheless, the potential benefits were inferred from the studies on patients with COPD, ILD, and PAH [13]. Treatment of OSA in patients with PH was associated with increased exercise capacity in those who underwent cardiac rehabilitation [41].

### 5.6. Weight Management

Obesity is likely a modifiable factor; moreover, weight loss likely benefits patients with both OSA and PH through a variety of mechanisms. Improvement in pulmonary hemodynamics was observed in patients with obesity who underwent bariatric surgery [65,66,67] and was accompanied by a reduction in several inflammatory markers [67].

Pharmacological weight management has also gained popularity. Patients with obesity treated with Glucagon-like peptide-1 receptor agonists (GLP-1 RAs) are associated with improved cardiac and endothelial function [68]. A GLP-1 RA with GIP (glucose-dependent insulinotropic polypeptide), tirzepatide, has been approved for moderate-to-severe OSA with obesity due to its efficacy in reducing apnea severity, achieving sleep apnea resolution (AHI < 5 or AHI 5–14 without excessive daytime sleepiness) and weight reduction [69].

### 5.7. Treatment of Volume Overload

Patients with PH often suffer from volume overload caused by both right heart dysfunction and occasional comorbid left-sided heart disease, which is common in afflicted patients [70]. Fluid overload can worsen OSA severity, augment its negative effects, and can potentially lead to decompensation in patients with pulmonary hypertension [71]. OSA can also contribute to peripheral edema, especially in patients with underlying obesity or impaired pulmonary function [72]. Diuretic therapy is recommended in patients with PH showing signs of fluid retention [42]. Nevertheless, diuretics alone might not be adequate for alleviating sleep apnea in all comers. An observational study using propensity score matching from France found that diuretics were associated with reduced OSA severity only in subgroups of patients—those who were overweight, moderately obese, or had a history of hypertension [73]. Limited experimental studies have shown only a slight reduction in the apnea–hypopnea index of 14–16% in patients using combination diuretic regimens (spironolactone/furosemide and spironolactone/metolazone) [74,75]. In addition, these studies were conducted in limited populations, with Fiori et al. [74] including only men with severe OSA and Kasai et al. [75] focusing on patients with moderate-to-severe OSA with uncontrolled hypertension, which limits the generalizability of these data.

Besides diuretic therapy, optimizing cardiac function is also a cornerstone of volume management. The use of sodium-glucose cotransporter 2 inhibitors (SGLT-2i) is now of particular interest in various patient subgroups with cardiac dysfunction [76]. There are also some reports mentioning improvement of OSA with SGLT-2i, albeit in a small number of subjects and limited to patients with diabetes [77,78]. The potential mechanisms of SGLT-2i in sleep apnea were recently reviewed [79]. Moreover, a study in animal models showed promising results supporting the benefit of SGLT-2i in PAH [80]. Further investigation of the clinical effect of SGLT-2i in patients with PH will be intriguing. Other potential interventions to alleviate fluid retention, such as restriction of sodium or increased exercise, are worth considering for inclusion in the management of volume status.

## 6. Conclusions

OSA influences pulmonary hemodynamics and prognosis in patients with WHO Group 3 PH. Primarily, these patients are prone to severe increases in PAP from the combination of baseline hypoxemia and/or hypercapnia, along with abnormalities induced by respiratory events during sleep. Whether isolated OSA induces pulmonary vascular changes and impairs the right ventricular function to a degree that affects the trajectory of diurnal pulmonary hemodynamics is still debatable. However, when clinicians evaluate patients with PH or OSA, they should be aware of the possibility that both conditions could contribute to patient symptoms and provide holistic management, depending on the primary causes of PH and other comorbidities. Positive airway pressure therapy, oxygen therapy, and weight management are the main therapies to be considered in these patients, with varying benefits depending on their contribution to pulmonary hemodynamics. Newer pharmacotherapies show promising results via multiple pathways and will potentially benefit this group of patients in the future.

## Figures and Tables

**Table 1:** Subclassifications of Group 3 pulmonary hypertension as defined by 6th and 7th WSPH expert consensus.

6th WSPH Group 3 Pulmonary Hypertension [10]	7th WSPH Group 3 Pulmonary Hypertension [14]
Obstructive lung disease or emphysema Restrictive lung disease Lung diseases with mixed restrictive/obstructive patterns Hypoxia without lung diseases Developmental lung disorders	COPD and/or emphysema Interstitial lung disease Combined pulmonary fibrosis and emphysema Other parenchymal lung diseases Nonparenchymal restrictive diseases: - Hypoventilation syndromes - Pneumonectomy Hypoxia without lung disease (e.g., high altitude) Developmental lung diseases

Abbreviations: World Symposium on Pulmonary Hypertension (WSPH); chronic obstructive lung disease (COPD).

**Table 2:** Treatment strategies to consider for obstructive sleep apnea and WHO Group 3 pulmonary hypertension.

Obstructive sleep apnea
Positive airway pressure therapy
Weight management (medical, surgical, lifestyle) Treatment of volume overload
WHO Group 3 PH
Optimize treatment of primary pulmonary diagnoses Long-term oxygen therapy if severe daytime hypoxemia Inhaled Treprostinil with PH-ILD
Pulmonary/cardiac rehabilitation Lung transplantation Weight management (medical, surgical, lifestyle)
Treatment of volume overload

Abbreviations: WHO: World Health Organization, PH: pulmonary hypertension.
